# Transfusion of Blood in Obstetrical Practice

**Published:** 1861-07

**Authors:** 


——Prof. Martin continues to meet with success in Transfusion of Blood in Obstetrical Practice. His last reported case was a primipara, æt 20, eight months gone, very anæmic from intra-uterine hæmorrhage, child dead, liquor amnion gone, and after a fruitless attempt at relief by tampon and stimulants, during which the fundus rose higher and higher; pulse became imperceptible; countenance fell; swooning ensued; temperature of body sunk, transfusion was decided on. Blood was taken from the median vein of a strong, healthy man-servant. After laying bare, by a cutaneous incision 4–5 lines long, the median vein of the right arm of the woman, the flat trocar was introduced, and the blood of the man, as it ran into a cup placed in water of about 100° F. temperature, was injected with the syringe, previously warmed with water of the same temperature, at four times, in the quantity of about six or seven ounces. The patient complained of no disagreeable sensation; some color returned to the cheeks, and uterine pains became active. On removal of the tampon, the os was found pretty well dilated, and the child was extracted with forceps, over two pounds of black grumous coagula coming away with the afterbirth.

After giving for refreshment a little champagne, a post-part. flooding ensued, which, though stopped by injections of diluted, first vinegar, and afterwards liq. ferri, sesquichloridi, caused such a degree of bloodlessness, that in view of the impossibility of hematosis by food or medicine, another transfusion of three ounces into the basilic vein of the right arm was made. Gradually the patient reacted, left her bed on the fourteenth day, and made a perfect recovery.

Dr. Martin adds to the report of this case the account of another in which he was called on to perform transfusion, after serous effusions into the pleural cavities had already taken place. He declined operating, however, and states his conviction that transfusion offers no hope after the secondary changes, in consequence of loss of blood, and especially serous effusions in the serous cavities of the chest and skull, have already occurred.

